# Is the Rise in Reported Dementia Mortality Real? Analysis of Multiple-Cause-of-Death Data for Australia and the United States

**DOI:** 10.1093/aje/kwac047

**Published:** 2022-03-11

**Authors:** Tim Adair, Jeromey Temple, Kaarin J Anstey, Alan D Lopez

**Keywords:** Australia, cardiovascular disease, causes of death, dementia, mortality, United States, vital statistics

## Abstract

Official statistics in Australia and the United States show large recent increases in dementia mortality rates. In this study, we assessed whether these trends are biased by an increasing tendency of medical certifiers (predominantly physicians) to report on the death certificate that dementia was a direct cause of death. Regression models of multiple-cause-of-death data in Australia (2006–2016) and the United States (2006–2017) were constructed to adjust dementia mortality rates for changes in death certification practices. Compared with official statistics, the recent increase in adjusted age-standardized dementia death rates was less than half as large in Australia and about two-thirds as large in the United States. Further adjustment for changes in reporting of dementia anywhere on the death certificate implied even lower increases in dementia mortality. Declines in reporting of cardiovascular diseases as comorbid conditions also contributed to rises in dementia mortality rates. The increasing likelihood of dementia’s being reported as directly leading to death largely explains recent increases in dementia mortality rates in both countries. However, studies have found that reported dementia on death certificates remains low compared with clinical evaluations of its prevalence. Improved guidance and training for certifiers in reporting of dementia on death certificates will help standardize mortality statistics within and between countries.

## Abbreviations

ASDR: age-standardized death rate
CVD: cardiovascular disease
GBD: Global Burden of Disease
ICD-10: *International Classification of Diseases, Tenth Revision*
MCOD: multiple cause of death
UCOD: underlying cause of death

In Australia and the United States, dementia will become an even more important public health issue in coming decades because of continued population aging (1). Accurate dementia mortality data enable estimation of how long a person with the condition can be expected to live and for how many of those years they will live with severe disability, and so are an important source of health intelligence to better inform clinical understanding of dementia and planning by various authorities for health and care services. Official dementia mortality statistics, collected by national authorities for all registered deaths in a population, provide valuable evidence with which to estimate the burden of disease from dementia and associated economic costs, to obtain a nuanced understanding of differentials by demographic and socioeconomic characteristics, and to identify conditions that are commonly comorbid with dementia mortality (2). As a consequence, there has been increasing interest by the public health community in obtaining more reliable and comparable data on the burden of dementia (3).

Official mortality statistics in high-income countries show that mortality rates from Alzheimer disease and other forms of dementia have been increasing in recent years (4–7). In Australia between 2010 and 2019, the reported number of dementia deaths increased by 67% and the dementia age-standardized death rate (ASDR) rose by 27%, with dementia now reported to be the leading cause of death for females and the second-leading cause for males (6). In the United States between 2009 and 2018, the number of dementia deaths rose by 51% and the dementia ASDR increased by 23%, with dementia now being the leading cause of death for US females and the third-leading cause for males (8). Based on these trends, the Australian Bureau of Statistics has predicted that dementia will be the leading cause of death for both sexes in Australia in coming years (9).

These reported trends in dementia mortality rates contrast with studies suggesting that dementia incidence and prevalence are either stable or decreasing (10–14). In an analysis of 7 population-based cohort studies conducted in Europe and North America, Wolters et al. (13) reported a 13% decennial decline in dementia incidence rates over the past 25 years, faster for males than for females. However, in another review of studies, Prince et al. (10) concluded that trends in prevalence are less clear because declines in incidence may be offset by improvements in survival. In Australia, there is less evidence on trends in dementia incidence and prevalence, with one study finding marginal declines in prevalence in recent years, standardized for the aging of the population, both for people accessing long-term care and for people using home care (15–18). Projections of dementia prevalence and associated economic costs generally assume that prevalence will remain constant in the future (19–22).

One possible reason for the divergent trends between official dementia mortality statistics and dementia incidence and prevalence is changes in medical certification practices. Dementia mortality reported in official statistics in high-income countries is based on what the medical certifier (predominantly physicians, but the certifier can be a coroner or, in the United States, a nurse practitioner or another designated agent, depending on the state) reports about causes of death on the International Form of Medical Certificate of Cause of Death (i.e., the international death certificate—see Web Figure 1, available at https://doi.org/10.1093/aje/kwac047) (23). Dementia can be reported either as leading directly to death (part 1 of the certificate) or as another significant condition contributing to death (part 2 of the certificate). This information is used, with *International Classification of Diseases, Tenth Revision* (ICD-10) coding rules (23), to select an underlying cause of death (UCOD), which is the information on mortality reported in official statistics.

For dementia to be reported on the death certificate, the decedent must 1) have had dementia, 2) have been diagnosed with dementia, and 3) have had dementia assessed by the medical certifier to have either led directly to death or been another significant condition that contributed to death. Studies suggest that dementia prevalence trends are essentially stable, or at least not increasing anywhere near the extent of dementia mortality according to official statistics. In this context, trends in dementia mortality may be affected by changes in the tendency of medical certifiers to report dementia anywhere on the death certificate, as well as whether they report dementia in part 1 or part 2. These changes may be due to improved diagnostic practices for dementia, shifting diagnostic preferences or “diagnostic fashions,” improved recognition and knowledge, and better-informed attitudes about dementia’s role in leading to death (20, 24). For example, 2 studies from the United Kingdom showed that reporting of dementia anywhere on a death certificate has increased substantially in recent decades, with one study (25) finding that the prevalence of any mention of dementia on the death certificate increased from 40% of decedents with dementia in 2006 to 63% in 2013 (25, 26). Underreporting of dementia on the death certificate has been found to be significant as compared with clinical evaluations in the United States (2 studies specifically focused on Alzheimer disease (27, 28)) and estimates from administrative data linkage studies from the Netherlands and Australia (16, 27–29). This reflects the underdiagnosis of dementia more generally, with one systematic review and meta-analysis finding that most dementia is undetected (30). Various studies have also found that dementia is more likely to be reported on a death certificate if it is more severe or if the deceased lived in an institution, was older, or had Alzheimer disease, and less likely if the deceased died in a hospital (25, 26, 31).

Trends in dementia mortality statistics are also influenced by changes in the number and type of comorbid conditions present at or around the time of death, which are especially common among the very old, and whether medical certifiers report these in part 1 or part 2 of the death certificate. For example, according to ICD-10 coding rules, when ischemic heart disease and dementia are both reported in part 1 of the death certificate, ischemic heart disease is selected as the UCOD. However, if ischemic heart disease is reported in part 2 and dementia in part 1, then dementia is selected as the UCOD. Differing certification practices can lead to substantial variations in dementia mortality rates; for example, dementia death rates at age 85 years or more in Australia are 6 times higher than those in Japan, despite very little difference in prevalence between the 2 countries (32, 33). Certification of deaths where there are multiple comorbid conditions, with often complex interactions, is challenged by their reliable diagnosis and also accurate attribution of the death to a chain of causes.

These measurement issues make comparisons of official dementia mortality statistics over time challenging. To develop improved estimates of trends in dementia mortality in Australia and the United States, we analyzed multiple-cause-of-death (MCOD) data, which comprise all conditions reported on death certificates for all registered deaths (i.e., the data used in official statistics). MCOD data are an underutilized data source which are particularly useful for better understanding of the sequential role of morbid conditions in leading to death, and so are particularly relevant for dementia. The specific objectives of our study were to:

$ \bullet $

develop and apply statistical models to estimate age-standardized dementia death rates and their trends in Australia and the United States, accounting for the likely bias arising from changing death certification practices; and

$ \bullet $

analyze the presence of comorbid conditions reported on the death certificate and how they influence trends in dementia mortality rates.

This additional intelligence should help to inform interpretation of data on dementia mortality, including evaluation of reported statistics on the comparative importance of dementia as a cause of death in Australia and the United States, and potentially elsewhere.

## METHODS

We used MCOD data for deaths occurring in Australia between 2006 and 2016 and in the United States between 2006 and 2017. Australian MCOD data for all registered deaths were available from the Australian Cause of Death Unit Record File (34). Population data by year, sex, and 5-year age group (up to age ≥95 years) were obtained from the Australian Bureau of Statistics (35). US MCOD data were obtained from the National Center for Health Statistics Multiple Cause of Death data file (36). US population data by year, sex, and 5-year age group (up to ≥95 years) were taken from the Global Burden of Disease (GBD) Study, because official population estimates did not provide data for specific age groups above 85 years (37, 38).

For each death, MCOD data contain all causes reported on the death certificate. We restricted our analyses to deaths of people aged 50 years or more who had dementia reported anywhere on the death certificate (either part 1 or part 2). We defined dementia as any of the following ICD-10 codes: F00 and G30 (Alzheimer disease), F01 (vascular dementia), G31 (other specified dementias, such as frontotemporal dementia or Lewy body dementia), and F03 (unspecified dementia). Our analyses included 2 measurements of dementia mortality:

$ \bullet $

dementia MCOD, where dementia was reported anywhere on the death certificate, and

$ \bullet $

dementia UCOD, where dementia was the UCOD.

We calculated ASDRs for dementia MCOD and dementia UCOD by sex and year, as reported numbers of deaths per 100,000 population; these were standardized to the population age distribution of Australia (both sexes) in 2006 to adjust for population aging in each country. We measured trends in the percentage of dementia MCOD deaths where either dementia or a cardiovascular disease (CVD) (ICD-10 codes I00–I99) was specified as the UCOD and trends in the percentage of dementia MCOD deaths where dementia and several other causes were reported in either part 1 or part 2 of the death certificate. The ICD-10 codes for these other causes are given in Web Table 1. These other causes were reported for 98% of dementia MCOD in Australia and 97% in the United States, excluding deaths where dementia was the only cause reported.

To adjust trends in the ASDR for dementia as the UCOD that are likely to have arisen, at least in part, from changing death certification practices, we fitted a multivariable logistic regression model for each sex in each country. Each of these regression models included all deaths for which dementia was reported on the death certificate (i.e., dementia MCOD). The models each had a dependent variable of dementia UCOD and included covariates representing factors expected to predict dementia’s being the UCOD. The coefficients from these regression models were used to predict the probability that each death was dementia UCOD, by using the regression coefficient for the most recent year of death (i.e., 2016 in Australia, 2017 in United States) to assume that the quality of dementia UCOD reporting has improved over time. The predicted probabilities were used to calculate adjusted dementia UCOD ASDRs by sex. Further details of the methods are shown in the Web Appendix.

The trends in adjusted dementia UCOD ASDRs would be influenced by changes in the dementia MCOD ASDR, which comprises an increasing proportion of deaths among people with dementia (25). We calculated adjusted dementia UCOD ASDRs assuming that the dementia MCOD ASDR had remained at the level of the most recent year throughout the entire period. This is consistent with evidence suggesting that the prevalence of dementia in each country has been stable. For example, in Australia in 2009, this is calculated as the adjusted dementia UCOD ASDR in 2009 divided by the dementia MCOD ASDR in 2009 multiplied by the dementia MCOD ASDR in 2016. Any trends in adjusted dementia UCOD ASDRs assuming constant MCOD ASDRs would then be attributable to changes in the reporting of each other cause on the death certificate.

Ethical approval for the study was provided by the University of Melbourne Medicine and Dentistry Human Ethics Sub-Committee (Melbourne, Victoria, Australia).

## RESULTS

**Figure 1:**
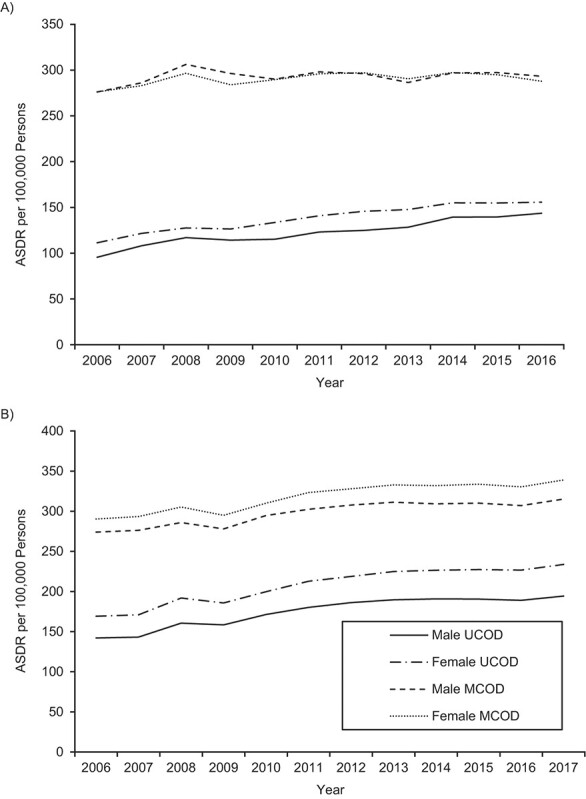
Age-standardized death rates (ASDRs) (number of deaths per 100,000 persons) for deaths with dementia reported as an underlying cause of death (UCOD) and dementia reported as one of multiple causes of death (MCOD) among individuals aged ≥50 years, by sex, Australia (2006–2016) (A) and United States (2006–2017) (B). Death rates were age-standardized to the 2006 Australian population of both sexes.

From 2006 to 2016, Australia’s dementia UCOD ASDR increased by 4.2% per year for males and 3.4% per year for females; meanwhile, the dementia MCOD ASDR increased much more modestly: 0.6% per year for males and 0.4% for females (Figure 1A). In the United States, from 2006 to 2017 the increase in the dementia UCOD ASDR was slower for males (2.9%/year) and comparable (3.0%/year) for females, while the dementia MCOD ASDR rose faster than in Australia at 1.3% per year for males and 1.4% per year for females (Figure 1B). The dementia MCOD ASDRs were 10%–20% higher in the United States than in Australia, while UCOD ASDRs were 35%–50% higher in the United States.

**Figure 2:**
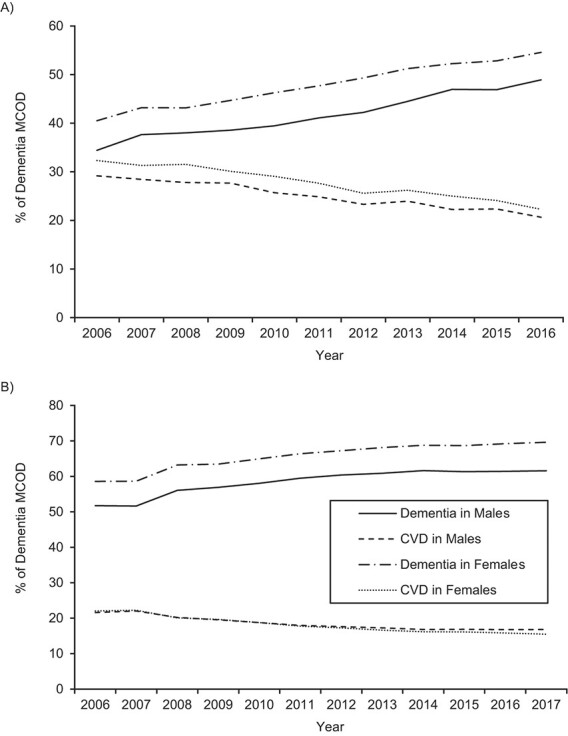
Age-standardized death rates (ASDRs) (number of deaths per 100,000 persons) for deaths with dementia reported as an underlying cause of death and cardiovascular disease (CVD) reported as an underlying cause of death (percentage of dementia multiple-cause-of-death (MCOD) cases) among persons aged ≥50 years, by sex, Australia (2006–2016) (A) and United States (2006–2017) (B).

CVD is the condition most commonly reported to be comorbid with dementia. Figure 2A indicates that at the same time there has been a sharp increase in the percentage of dementia MCOD deaths in Australia where dementia is the underlying cause, there has been a corresponding decline in the percentage where CVD is the underlying cause. A similar trend is apparent in the United States, though less extreme (Figure 2B). Notably, at both the beginning and the end of the study period, dementia as an underlying cause comprised a higher percentage of deaths in the United States than in Australia, and CVD as an underlying cause comprised a lower proportion. As expected, these trends corresponded to trends in the reporting of these causes in either part 1 or part 2 of the death certificate (Web Figure 2). In Australia, the rate of reporting of dementia in part 1 of the death certificate (as a percentage of dementia MCOD) increased over the study period, reporting of CVD in part 1 declined, and reporting of CVD in part 2 remained steady. In the United States, the rate of reporting of dementia in part 1 of the death certificate also increased, but not as quickly as in Australia, and reporting of CVD in part 1 declined only slightly and in part 2 remained steady. Changes in reporting of all causes of death as a percentage of dementia MCOD are shown in Table 1 and Web Table 1. In both countries, there has been a sharp decline in pneumonia reported in part 1. On the other hand, the percentage of dementia MCOD comprised by each other cause is similar for each country and has remained relatively constant over the period.

**Table 1:** Selected Causes of Death Reported in Part 1^a^ or Part 2^b^ of the International Death Certificate (Percentage of Deaths That Have Dementia Reported As One of Multiple Causes of Death) Among Persons Aged ≥50 Years, by Sex, Australia (2006–2016) and United States (2006–2017)

	**Country, Sex, and Year**							
	**Australia**				**United States**			
	**Male**		**Female**		**Male**		**Female**	
**Cause of Death** ^**c**^ **and** **Part of Death Certificate**	**2006**	**2016**	**2006**	**2016**	**2006**	**2017**	**2006**	**2017**
Ischemic heart disease								
1	16.9	11.9	17.1	10.7	13.9	9.6	12.8	7.6
2	9.2	10.7	6.3	6.3	7.4	7.4	4.9	4.3
Stroke								
1	15.6	11.4	18.1	12.9	7.5	6.8	7.6	7.0
2	6.6	5.9	4.8	4.4	4.7	3.8	3.9	3.2
All cancers								
1	8.4	7.9	5.4	5.2	5.2	4.2	3.3	2.7
2	6.4	6.5	3.3	3.3	4.0	3.2	2.0	1.6
Pneumonia								
1	26.0	16.9	21.6	13.9	12.4	6.1	8.9	4.2
2	0.9	0.3	0.5	0.3	1.2	0.9	0.9	0.6
Chronic respiratory disease								
1	4.7	3.7	2.1	2.1	4.6	3.5	2.9	2.8
2	3.8	4.4	1.9	2.4	4.7	4.4	2.7	2.9
Other respiratory diseases								
1	16.1	19.0	10.4	12.8	14.4	12.3	9.7	8.7
2	2.3	2.0	1.6	1.9	1.4	1.5	1.0	1.1
Ill-defined causes								
1	12.4	14.7	14.7	16.6	17.4	17.5	19.3	19.1
2	2.9	3.8	3.0	3.9	4.9	6.5	4.9	6.4

^a^ All conditions and diseases that directly lead to death.

^b^ All other significant conditions contributing to death.

^c^ Results for all causes are shown in Web Table 1.

Univariate and bivariate results are shown in Web Tables 2 and 3, and logistic regression results are presented in Web Tables 4 and 5. The coefficients for each cause and sex are consistent between the 2 countries. There are negative coefficients for the reporting in part 1 of CVDs, especially ischemic heart disease and stroke, chronic kidney disease, cancers, chronic respiratory disease, Parkinson disease, and injuries. That is, where these causes are reported in part 1, the probability of dementia being the underlying cause is reduced. Positive coefficients are found for pneumonia, urinary tract infections, and ill-defined causes where reported in part 1. The coefficients increase with age, and in Australia the coefficients are larger in the most recent year of death than in the United States, implying a sharper increase in the probability of dementia being the underlying cause, controlling for all other variables.

**Figure 3:**
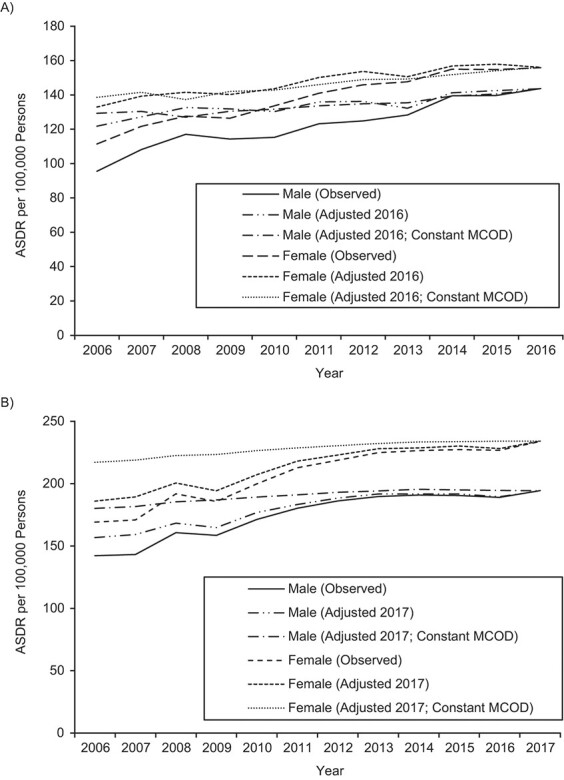
Age-standardized death rates (ASDRs) (number of deaths per 100,000 persons) for deaths with dementia reported as an underlying cause of death among persons aged ≥50 years, observed, adjusted to the most recent year (2016 in Australia, 2017 in the United States), and adjusted to the most recent year while assuming constant dementia multiple-cause-of-death (MCOD) ASDRs, by sex, Australia (2006–2016) (A) and United States (2006–2017) (B).

In Australia, when the dementia UCOD ASDRs were adjusted for changing certification practices, the annual rate of increase was substantially slower than reported, for both males (adjusted 1.7%, observed 4.2%) and females (adjusted 1.6%, observed 3.4%) (Figure 3A, Table 2). That is, the 2006 ASDRs were higher than observed when adjusted for 2016 certification practices. Assuming a constant MCOD ASDR over the period, the dementia UCOD ASDR increased for both males (1.0%) and females (1.2%), but at a slower rate than when just adjusting for changing certification practices; even so, these results imply much more modest 11% and 13% increases in the dementia UCOD ASDR from 2006 to 2016 for males and females, respectively, compared with 51% and 40% increases as suggested by official statistics.

**Table 2:** Annual Rate of Change (%) in the Age-Standardized Rate of Deaths With Dementia Reported as an Underlying Cause of Death—Observed, Adjusted to 2016/2017 Certification Practices, and Adjusted to 2016/2017 Certification Practices While Assuming a Constant Age-Standardized Death Rate for Deaths With Dementia Listed as One of Multiple Causes of Death—Among Persons Aged ≥50 Years, by Sex, Australia (2006–2016) and United States (2006–2017)

	**Country and Sex**			
	**Australia**		**United States**	
**Type of Dementia UCOD ASDR**	**Male**	**Female**	**Male**	**Female**
Observed	4.2	3.4	2.9	3.0
Adjusted to 2016/2017	1.7	1.6	2.0	2.1
Adjusted to 2016/2017, assuming a constant dementia MCOD ASDR	1.0	1.2	0.7	0.7

Abbreviations: ASDR, age-standardized death rate; MCOD, multiple cause of death; UCOD, underlying cause of death.

In the United States, where the rate of increase in the observed dementia UCOD ASDR was lower than in Australia, after adjusting for changing certification practices, these figures were in fact higher than in Australia (adjusted 2.0% and observed 2.9% for males; adjusted 2.1% and observed 3.0% for females) (Figure 3B). When also assuming a constant MCOD ASDR from 2006 to 2017, the dementia UCOD ASDR increased by 0.7% for both males and females, slower than in Australia, because the rate of increase in the MCOD ASDR was quicker in the United States. This implies an 8% increase in the dementia UCOD ASDR from 2006 to 2017 for both sexes—much lower than the observed increase of 37% for males and 38% for females.

By standardizing to 2006 death certification practices, dementia in Australia would have been ranked as the fifth-leading cause of death in males (rather than second in official statistics; a 20% lower ASDR) and the third-leading cause of death in females (rather than first; a 15% lower ASDR) (Table 3). For the United States, standardizing to 2006 death certification practices implies that dementia would be ranked fourth in males, rather than third (an 8% lower ASDR), while for females it would still be ranked first (but with an 8% lower ASDR).

**Table 3:** Rank and Percent Differences in the Age-Standardized Rate of Deaths With Dementia Reported as an Underlying Cause of Death, Observed and Adjusted to 2006 Certification Practices, Among Persons Aged ≥50 Years, by Sex, Australia (2016) and United States (2017)

	**Country and Sex**							
	**Australia**				**United States**			
	**Male**		**Female**		**Male**		**Female**	
**Type of Dementia** **UCOD ASDR**	**LCOD** **Rank**	**%** **Difference** ^**a**^	**LCOD** **Rank**	**%** **Difference**	**LCOD** **Rank**	**%** **Difference**	**LCOD** **Rank**	**%** **Difference**
Observed	2	NA	1	NA	3	NA	1	NA
Adjusted to 2006	5	−19.7	3	−14.8	4	−8.1	1	−8.1

Abbreviations: ASDR, age-standardized death rate; LCOD, leading cause of death; NA, not applicable; UCOD, underlying cause of death.

^a^ Percent difference in the dementia UCOD ASDR (adjusted vs. observed).

## DISCUSSION

Our study shows that the reported rise in dementia mortality rates in Australia and the United States, based on official statistics, has been far less rapid than implied by the data, primarily due to changes in certification practices when reporting dementia on the death certificate. In Australia, the annual increase in dementia mortality rates, after adjusting just for changing certification practices, is likely to have been less than half that observed from vital statistics, while in the United States it is about one-third lower. After also adjusting trends in mortality rates by assuming that reporting of dementia as an MCOD has been constant, we estimate that dementia mortality rates in Australia increased at an even slower rate. The increases in dementia mortality, after adjustments, are contributed to by changes in the composition of comorbid conditions reported along with dementia, particularly the decline in reporting of CVD.

The most relevant alternative analysis of dementia mortality trends was conducted in the GBD Study 2019, where investigators estimated that rates of dementia mortality in Australia rose only by 0.4% per year for males between 2006 and 2019 and were unchanged for females, while in the United States rates declined for both males and females, by 0.2% per year (39). The GBD Study approach was based upon excess all-cause mortality for people with dementia and measurement of the proportion of those excess deaths that have dementia as an underlying cause, based on linked mortality and inpatient records and clinical markers of severe end-stage dementia, and overall was a stricter measure of dementia mortality than that employed in our study (39). For Australia, our findings based on an MCOD analysis imply annual rates of increase since 2006 that are 0.6 and 1.2 percentage points higher than those estimated by the GBD Study 2019 for males and females, respectively, while for the United States our results suggest an annual rate of increase that is 0.9 percentage points higher for each sex.

In our view, the principal explanation for our findings relates to changes in certification practices for dementia mortality in Australia and the United States in recent years; that is, dementia is being more commonly reported as a cause directly leading to death rather than another condition contributing to death. The increased reporting of dementia mortality is consistent with 2 UK studies and may reflect improvements in the understanding of dementia as a cause of death, compared with the low reporting identified in the United States in the 1990s, when only one-quarter of deaths of people with a clinical diagnosis of dementia had the condition reported anywhere on the death certificate (25, 26, 40). It is likely that the factors driving improvements in the reporting of dementia anywhere on the death certificate would also explain the increased reporting of dementia as an underlying cause found in our study. A factor that may have contributed to increased reporting of dementia is improved diagnostic practices involving assessment of medical history, physical examination, and evaluation of cognitive abilities, as well as the use of laboratory tests and brain scans (41). Dementia remains underreported on death certificates in comparison with clinical evaluations of dementia prevalence, which reflects the underdiagnosis of dementia more generally (25, 27, 28, 30). This issue needs to be borne in mind when interpreting this study’s results. We were unable to measure more closely any changes in the likelihood of dementia’s being reported on the death certificate of a decedent who had been diagnosed with dementia. However, it remains unclear whether the reporting of dementia as the UCOD will continue to increase in the future or if an upper limit will be reached. For example, in an analysis of linked inpatient and mortality data, the GBD investigators concluded that dementia would not be the UCOD for a significant proportion of decedents who had dementia (39).

The continued increase in dementia mortality rates, even after adjusting for changing certification practices and assuming constant dementia MCOD rates, is explained by changes in the composition of reporting of other causes on the death certificate and, in particular, decreased reporting of CVD in part 1 of the death certificate. Our modeling demonstrates that reporting of dementia as an underlying cause is less likely if a CVD is reported in part 1 of the death certificate. CVD mortality has declined substantially in high-income countries over the past half-century, although these declines have recently slowed in many countries, and rates are now even rising in the United States, especially in younger cohorts (42). This suggests that changes in the etiology of dementia mortality, away from CVD, have acted to increase reporting of dementia as a UCOD. There was also a decline in reporting of pneumonia in part 1, which increases the probability of dementia being the underlying cause, but not to the same extent that CVDs decrease the probability, and hence this only slightly offsets the impact of lower reporting of CVD. While there are challenges with accurately measuring comorbid conditions and attributing a death to a chain of causes, MCOD data reveal insights into how trends in comorbid conditions can interact with ICD-10 coding rules to influence reported trends in dementia mortality.

Our study used statistical modeling to adjust trends in dementia mortality in Australia and the United States, but we did not attempt to directly compare levels of dementia mortality between the 2 countries. Based on the most recent year of data available, the United States had higher dementia mortality rates than Australia when measured as a UCOD, mostly due to the United States having a higher proportion of deaths with dementia reported as the UCOD on the death certificate. Interestingly, the GBD investigators estimate that dementia mortality rates are 5% higher in Australia than in the United States for males, and 7% higher for females. While it is apparent that reporting of dementia has improved over time, there remain problems with how dementia is reported by certifiers as a cause of death, with a substantial proportion of dementia UCOD deaths being reported as unspecified. Although volume 2 of the ICD-10 lists dementia as an ill-defined term and instructs certifiers to use more specific terms, such as Alzheimer disease, improved guidance for and better training of physicians on this topic would standardize reporting of dementia mortality and improve the comparative assessment of dementia mortality trends (23). ICD-10 coding rules have also been implemented to select vascular dementia as the underlying cause if unspecified dementia is reported in part 1 with stroke; however, this cause comprises only 10% of dementia UCOD deaths in Australia and 6% in the United States (34). Improved reporting of vascular dementia would potentially reveal whether mortality from this cause is declining, consistent with CVD mortality. Our models were also unable to include information on the place of death (shown by one study (26) to predict the presence of dementia on the death certificate) since this information was not available for both countries and is generally not specified. Improvements in reporting and diagnosis of the type of dementia could improve these models and thus the measurement of dementia mortality trends.

Nonetheless, the ability of the methods to effectively exploit MCOD data to better understand how trends in dementia mortality rates are influenced by changing certification practices, as well as by changes in the composition of comorbid conditions, suggests that their application to MCOD data for other countries would improve our global understanding of dementia mortality trends. The methods presented in this study could also be applied to other causes of death that are commonly reported with numerous comorbid conditions, such as diabetes and CVD, to improve understanding of how trends in comorbid conditions can interact with ICD-10 coding rules to influence mortality trends. The routine application of statistical methods, such as those we have applied, to reported dementia mortality data would improve their policy utility through a better understanding of the likely impact of certification practices on leading causes of death, as well as the role of compositional effects. In particular, as populations in many countries continue to age, a more reliable understanding of dementia mortality patterns and trends, based on official statistics, will sharpen our knowledge about the true overall burden and distribution of dementia mortality in populations, increase understanding of the role of comorbidity in leading to death, help estimate life expectancy and years lived with severe disability for a person with dementia, and more generally strengthen the policy utility of vital statistics to inform the planning and management of dementia, including intervention strategies to improve longevity.

## Supplementary Material

Web_Material_kwac047
