# Supplementary material for: Is the Rise in Reported Dementia Mortality Real? Analysis of Multiple-Cause-of-Death Data for Australia and the United States
Source: Am J Epidemiol. 2022 Mar 11;191(7):1270–9. doi: 10.1093/aje/kwac047 (PMC9247423; doi:10.1093/aje/kwac047)
Supplement: Web_Material_kwac047 [file web_material_kwac047.pdf]

WEB MATERIAL

Is the Rise in Reported Dementia Mortality Real? Analysis of Multiple-Cause-of-Death Data for Australia and the United States

Tim Adair, Jeromey Temple, Kaarin J. Anstey, and Alan D. Lopez

Table of Contents

Web Appendix

Web Table 1

Web Table 2

Web Table 3

Web Table 4

Web Table 5

Web Figure 1

Web Figure 2

WEB APPENDIX

Further Detail on Methods

To adjust trends in dementia underlying cause of death (UCOD) age-standardized dementia death rates (ASDR) due to likely bias arising from changing death certification practices, we firstly estimated a multivariable logistic regression model for each sex in each country. These regression models included all deaths where dementia was reported on the death certificate (i.e. dementia multiple cause of death (MCOD)) and had a dependent variable of the death being dementia UCOD. Each regression model had covariates of age at death, year of death, and whether each other cause of death (listed in Web Tables 1 and 2) was reported on the death certificate (either in Part 1 or Part 2). These covariates represent factors expected to predict the likelihood of dementia being the UCOD.

For each country and sex,

$$ln\left(\frac{p}{1-p}\right) = \beta_0 + \beta_1 Age_x + \beta_2 Year_y + \beta_3 Cause_{z1} + \ \dots + \beta_{19} Cause_{z17} \qquad (1)$$

where *p* is the probability that dementia is the UCOD (out of all dementia MCOD deaths), *Age* represents age at death (5-year age group *x*: 50-54 years, 55-59 … 95+), *Year* represents calendar year *y* of death, *Cause* represents whether cause of death *z* (representing each of 17 causes from *z1* to *z17*) is reported on the death certificate.

Other covariates suggested by the literature to predict whether dementia is the UCOD were not included because either they were not available in the datasets (place of death and severity of dementia) or could not be included because of data quality issues (type of dementia: Alzheimer's disease is under-reported as a UCOD because 60% of dementia UCOD deaths in Australia and 51% in the United States are 'unspecified' dementia) (15).

Using the coefficients from these regression models, we predicted the probability that each death is dementia UCOD by using, for all deaths, the year of death coefficient for the most recent year of data (i.e. 2016 in Australia, 2017 in the United States). This implicitly assumes that the regression coefficient measuring year of death captures changes in death certification practices over

the period; the most recent year was chosen on the assumption that the quality of dementia UCOD reporting has improved over time. All other coefficients were multiplied by the reported data for that covariate. Using Australia as an example:

$$ln\left(\frac{\hat{p}}{1-\hat{p}}\right) = \beta_0 + \beta_1 Age_x + \beta_{2,2016} Year_y + \beta_3 Cause_{z1} + \ldots + \beta_{19} Cause_{z17} \qquad (2)$$

where $\beta_{2,2016}$ is the coefficient for death in 2016 (2017 is used for the United States), $ln\left(\frac{\hat{p}}{1-\hat{p}}\right)$ was calculated directly from the regression equation and the inverse logit of this figure, $\hat{p}$, is the predicted probability that a death is dementia UCOD.

The predicted probability that each death is dementia UCOD ($\hat{p}$) was then summed to calculate total adjusted dementia UCOD deaths by age, sex, year and country, which was used to calculate adjusted dementia UCOD ASDRs by sex, year and country (per 100,000; standardized to the population age distribution of Australia for both sexes in 2006).

We also compared results from official statistics (i.e. unadjusted) in the most recent year of data to estimates of mortality derived by reversing the adjustment process above and assuming that death certification practices for dementia in 2006 applied (i.e. using a coefficient of 0 for year of death because 2006 is the reference category for this covariate).

References

1. Gao L, Calloway R, Zhao E, et al. Accuracy of death certification of dementia in population-based samples of older people: analysis over time. *Age Ageing* 2018;47(4):589-94.
2. Perera G, Stewart R, Higginson IJ, et al. Reporting of clinically diagnosed dementia on death certificates: retrospective cohort study. *Age Ageing* 2016;45(5):668-73.
3. Romero JP, Benito-Leon J, Louis ED, et al. Under Reporting of Dementia Deaths on Death Certificates: A Systematic Review of Population-based Cohort Studies. *J Alzheimers Dis* 2014;41(1):213-21.
4. Australian Coordinating Registry. Cause of Death Unit Record File, 2006-17. Brisbane: Australian Coordinating Registry; 2019.
5. National Center for Health Statistics. Mortality Multiple Cause Files, 2000-17. Hyattsville, MD: National Center for Health Statistics Centers for Disease Control; 2019.

Web Table 1. Causes Reported in Part 1 or Part 2 of Death Certificate (% of Dementia MCOD), by Sex, 50+ Years, Australia 2006-16 and US 2006-17

**Cause (ICD Codes)**	**Part**	**Australia**				**United States**			
		**Male**		**Female**		**Male**		**Female**	
		**2006**	**2016**	**2006**	**2016**	**2006**	**2017**	**2006**	**2017**
Dementia	1	40.4	53.1	43.8	56.9	57.0	67.1	61.8	72.8
	2	59.6	46.9	56.2	43.1	43.0	32.9	38.2	27.2
IHD (I20-I25)	1	16.9	11.9	17.1	10.7	13.9	9.6	12.8	7.6
	2	9.2	10.7	6.3	6.3	7.4	7.4	4.9	4.3
Stroke (I60-I69)	1	15.6	11.4	18.1	12.9	7.5	6.8	7.6	7.0
	2	6.6	5.9	4.8	4.4	4.7	3.8	3.9	3.2
Other heart disease (I26-I51)	1	16.5	15.3	18.0	16.3	19.2	17.2	19.8	17.1
	2	7.8	10.2	7.7	8.7	8.7	10.3	8.1	8.8
Hypertensive heart disease (I10-I13)	1	6.6	6.1	8.9	8.3	5.8	6.4	7.2	7.5
	2	6.3	7.8	8.0	9.6	10.4	12.6	11.7	13.1
Other CVD (I00-I09, I14-I19, I52-I59, I70-I99)	1	5.7	2.9	5.7	2.8	3.6	2.1	3.7	2.0
	2	2.7	2.1	2.0	1.4	2.7	2.4	2.2	1.9

Diabetes (E10-E14)	1	4.1	4.4	3.6	3.7	2.9	2.6	2.4	2.1
	2	6.3	8.7	4.6	6.3	7.3	7.9	5.9	5.8
Chronic kidney disease (N18)	1	2.6	2.8	1.4	2.2	1.5	2.5	0.9	1.8
	2	2.3	3.3	1.4	2.5	1.5	4.5	0.9	3.3
Other kidney disease (N17, N19)	1	6.1	4.8	4.7	5.0	3.6	2.3	2.8	1.8
	2	1.8	1.4	1.2	1.1	2.5	1.1	1.6	0.8
All cancers (C00-D44)	1	8.4	7.9	5.4	5.2	5.2	4.2	3.3	2.7
	2	6.4	6.5	3.3	3.3	4.0	3.2	2.0	1.6
Sepsis (A41)	1	5.6	5.6	4.5	4.3	4.8	4.7	4.0	3.6
	2	0.4	0.1	0.2	0.1	0.3	0.4	0.3	0.3
Pneumonia (J18)	1	26.0	16.9	21.6	13.9	12.4	6.1	8.9	4.2
	2	0.9	0.3	0.5	0.3	1.2	0.9	0.9	0.6
Chronic respiratory disease (J40-J44)	1	4.7	3.7	2.1	2.1	4.6	3.5	2.9	2.8
	2	3.8	4.4	1.9	2.4	4.7	4.4	2.7	2.9
	1	16.1	19.0	10.4	12.8	14.4	12.3	9.7	8.7

Other respiratory diseases (J00-J17, J19-J39, J45-J99)	2	2.3	2.0	1.6	1.9	1.4	1.5	1.0	1.1
Parkinson's disease (G20)	1	2.9	3.6	1.2	1.3	3.4	3.8	1.4	1.4
	2	4.3	3.2	1.9	1.6	2.8	2.2	1.3	0.9
Urinary tract infection (N39)	1	2.2	2.9	2.5	2.8	2.2	1.7	2.5	1.7
	2	1.0	0.9	1.6	1.1	1.0	0.9	1.3	1.1
Ill-defined (R00-R99)	1	12.4	14.7	14.7	16.6	17.4	17.5	19.3	19.1
	2	2.9	3.8	3.0	3.9	4.9	6.5	4.9	6.4
Injuries (V00-X44, X46-Y99)	1	4.6	5.5	3.6	4.4	3.3	2.9	2.2	2.1
	2	3.4	3.2	3.5	3.6	2.4	2.4	2.3	2.4

Abbreviations: CVD, cardiovascular disease; ICD, *International Classification of Diseases*; MCOD, multiple cause of death.

Web Table 2. Univariate and Bivariate Statistics (%), Australia, 50+ Years, 2006-16, Dementia MCOD With At Least One Other Condition Reported

Variable	Part	Male			Female		
		Univariate	Dementia UCOD	Dementia Not UCOD	Female	Dementia UCOD	Dementia Not UCOD
Dementia UCOD		39.7	-	-	44.5	-	-
**Cause**							
IHD	1	15.7	3.8	23.5	15.1	3.2	24.7
	2	10.5	12.1	9.5	7.1	8.2	6.2
Stroke	1	14.3	7.5	18.8	16.4	7.3	23.8
	2	6.5	5.7	7.0	5.1	4.7	5.4
Other heart disease	1	16.8	10.0	21.3	18.6	11.2	24.6
	2	9.6	9.4	9.7	8.9	9.0	8.9
Hypertensive heart disease	1	6.9	2.9	9.6	10.0	4.3	14.6
	2	7.7	8.1	7.4	9.7	10.9	8.8
Other CVD	1	4.3	1.7	6.0	4.4	1.7	6.6
	2	2.6	2.3	2.9	1.9	1.8	2.0

Diabetes	1	5.3	2.6	7.1	4.4	2.3	6.1
	2	7.5	8.0	7.2	5.8	6.3	5.5
Chronic kidney disease	1	2.8	1.3	3.8	2.3	1.1	3.2
	2	2.8	2.5	3.0	2.0	1.9	2.1
Other kidney disease	1	5.5	4.7	6.1	5.5	5.0	5.9
	2	1.6	1.5	1.7	1.4	1.4	1.3
All cancers	1	8.7	1.6	13.3	5.7	0.8	9.6
	2	6.8	6.7	6.9	3.6	3.8	3.5
Sepsis	1	5.9	5.2	6.5	4.8	4.0	5.4
	2	0.2	0.1	0.2	0.1	0.1	0.2
Pneumonia	1	21.9	32.8	14.7	19.2	29.0	11.3
	2	0.6	0.5	0.6	0.4	0.4	0.4
Chronic respiratory disease	1	4.4	1.2	6.4	2.4	0.6	3.8
	2	4.3	3.7	4.7	2.2	2.0	2.4
Other respiratory diseases	1	18.9	21.7	17.1	12.6	13.6	1.8
	2	2.1	2.0	2.2	1.8	1.7	1.9

Parkinson’s disease	1	3.6	0.7	5.5	1.5	0.3	2.4
	2	4.1	2.1	5.3	1.8	0.8	2.6
Urinary tract infection	1	2.8	3.8	2.2	2.9	4.1	1.9
	2	1.0	1.1	0.9	1.4	1.6	1.2
Injuries	1	4.7	5.9	2.8	3.5	2.1	4.7
	2	3.2	3.5	2.6	3.6	3.2	4.0
Ill-defined	1	14.4	18.9	11.5	16.7	22.8	11.8
	2	3.2	3.2	3.2	3.4	3.5	3.2
**Age**							
50-69		3.7	3.8	3.6	1.6	1.8	1.5
70-74		5.5	5.4	5.6	2.6	2.5	2.6
75-79		12.5	12.1	12.8	7.2	6.9	7.4
80-84		24.1	23.1	24.8	17.1	16.4	17.7
85-89		29.8	2..7	29.8	29.4	28.2	30.4
90-94		18.7	19.5	18.2	27.9	28.5	27.5
95+		5.8	6.5	5.3	14.2	15.7	12.9

**Year**							
2006		6.9	5.6	7.8	7.4	6.2	8.4
2007		7.5	6.7	8.1	7.9	7.0	8.5
2008		8.4	7.6	8.9	8.6	7.8	9.2
2009		8.4	7.7	8.9	8.4	7.8	8.9
2010		8.6	8.1	8.9	8.9	8.6	9.1
2011		9.2	9.0	9.3	9.3	9.3	9.4
2012		9.5	9.5	9.5	9.6	9.9	9.4
2013		9.6	10.2	9.2	9.6	10.2	9.1
2014		10.3	11.4	9.5	10.1	11.0	9.4
2015		10.7	11.8	10.0	10.1	11.0	9.4
2016		10.9	12.4	9.9	10.1	11.4	9.2
No.		86,835	-	-	142,281	-	-

Web Table 3. Univariate and Bivariate Statistics (%), United States, 50+ Years, 2006-17, Dementia MCOD With At Least One Other Condition Reported

Variable	Part	Male			Female		
		Univariate	Dementia UCOD	Dementia Not UCOD	Univariate	Dementia UCOD	Dementia Not UCOD
Dementia UCOD		51.1	-	-	57.5	-	-
**Cause**							
IHD	1	13.4	3.3	24.0	11.9	2.7	24.5
	2	9.1	9.5	8.7	5.7	5.9	5.6
Stroke	1	7.8	4.2	11.5	8.4	3.9	14.6
	2	4.6	4.1	5.7	4.3	3.8	5.0
Other heart disease	1	21.2	16.1	26.5	22.6	17.1	30.0
	2	11.3	10.3	12.4	10.7	9.8	12.0
Hypertensive heart disease	1	7.3	3.7	11.4	9.2	4.8	15.1
	2	14.2	13.5	14.8	16.2	16.1	16.3
Other CVD	1	3.2	1.7	4.7	3.4	1.7	5.6
	2	3.0	2.6	3.3	2.5	2.3	2.8

Diabetes	1	3.2	1.7	4.7	2.8	1.6	4.5
	2	9.1	8.2	10.0	7.4	6.8	8.3
Chronic kidney disease	1	2.4	1.5	3.3	1.7	1.1	2.6
	2	3.6	3.2	4.1	2.7	2.5	3.0
Other kidney disease	1	3.5	3.1	3.8	2.9	2.6	3.2
	2	2.1	1.8	2.4	1.5	1.3	1.8
All cancers	1	5.4	0.9	10.1	3.7	0.5	8.0
	2	4.1	3.8	4.4	2.2	2.1	2.5
Sepsis	1	5.4	5.1	5.7	4.5	4.1	5.1
	2	0.4	0.4	0.5	0.4	0.3	0.4
Pneumonia	1	10.2	13.4	6.9	7.7	9.7	5.1
	2	1.2	1.1	1.3	0.9	0.8	1.0
Chronic respiratory disease	1	4.7	1.4	8.2	3.6	1.0	7.1
	2	5.3	4.4	6.3	3.6	3.1	4.4
Other respiratory diseases	1	15.4	16.4	14.3	11.3	11.4	11.1
	2	1.7	1.6	1.9	1.4	1.2	1.6

Parkinson’s disease	1	4.1	0.6	7.9	1.7	0.3	3.7
	2	2.9	1.4	4.4	1.3	0.7	2.3
Urinary tract infection	1	2.2	2.7	1.6	2.5	3.2	1.6
	2	1.1	1.2	1.0	1.5	1.6	1.4
Injuries	1	3.5	2.5	4.6	2.5	1.6	3.8
	2	2.7	1.8	3.7	2.8	2.0	4.0
Ill-defined	1	22.2	30.0	14.0	26.1	34.6	14.7
	2	6.9	6.8	7.0	7.2	7.3	7.1
**Age**							
50-69		5.1	4.6	5.7	2.3	2.1	2.6
70-74		6.1	5.6	6.6	3.3	3.0	3.6
75-79		12.4	11.8	13.1	7.8	7.4	8.5
80-84		22.0	21.4	22.6	16.8	16.2	17.8
85-89		27.8	28.0	27.6	27.2	26.9	27.7
90-94		19.6	20.9	18.3	26.6	27.3	25.6
95+		6.9	7.7	6.1	16.0	17.2	14.3

**Year**							
2006		6.8	6.0	7.7	7.2	6.3	8.4
2007		7.0	6.1	8.0	7.4	6.5	8.6
2008		7.4	7.2	7.7	7.7	7.5	7.1
2009		7.3	7.1	7.5	7.5	7.3	7.9
2010		8.0	7.9	8.0	8.0	8.0	8.1
2011		8.4	8.6	8.2	8.5	8.7	8.3
2012		8.7	9.0	8.4	8.7	9.0	8.3
2013		9.0	9.3	8.6	8.9	9.3	8.4
2014		9.0	9.3	8.6	8.8	9.1	8.3
2015		9.3	9.6	8.9	9.0	9.3	8.5
2016		9.4	9.7	9.1	9.0	9.3	8.4
2017		9.9	10.2	9.5	9.3	9.8	8.7
No.		1,141,591	-	-	2,096,565	-	-

Web Table 4. Regression Results of Dementia UCOD, Australia

Variable	Part	Coefficient (95% Confidence Interval)	
		Male	Female
Cause (for each cause, Ref. is “not reported on MCCD”)			
IHD	1	-2.34 (-2.40 – -2.27)	-2.65 (-2.70 – -2.60)
	2	-0.08 (-0.13 – -0.02)	-0.12 (-0.17 – -0.07)
Stroke	1	-1.64 (-1.70 – -1.59)	-2.02 (-2.06 – -1.98)
	2	-0.68 (-0.75 – -0.62)	-0.67 (-0.72 – -0.61)
Other heart disease	1	-0.84 (-0.89 – -0.79)	-1.02 (-1.05 – -0.98)
	2	-0.33 (-0.39 – -0.27)	-0.37 (-0.41 – -0.32)
Hypertensive heart disease	1	-0.77 (-0.85 – -0.69)	-0.89 (-0.94 – -0.84)
	2	0.06 (0.00 – 0.12)	0.07 (0.03 – 0.12)
Other CVD	1	-1.24 (-1.34 – -1.14)	-1.32 (-1.40 – -1.25)
	2	-0.15 (-0.25 – -0.04)	-0.17 (-0.27 – -0.08)
Diabetes	1	-0.88 (-0.96 – -0.79)	-0.85 (-0.92 – -0.77)
	2	-0.04 (-0.10 – 0.02)	-0.03 (-0.08 – 0.03)
Chronic kidney disease	1	-1.23 (-1.35 – -1.11)	-1.35 (-1.44 – -1.25)
	2	-0.28 (-0.39 – -0.18)	-0.29 (-0.38 – -0.20)
Other kidney disease	1	-0.48 (-0.55 – -0.41)	-0.60 (-0.65 – -0.54)
	2	-0.27 (-0.40 – -0.14)	-0.07 (-0.18 – 0.05)
All cancers	1	-3.15 (-3.25 – -3.06)	-3.61 (-3.71 – -3.52)
	2	-0.36 (-0.42 – -0.30)	-0.24 (-0.31 – -0.17)
Sepsis	1	-1.00 (-1.07 – -0.93)	-1.22 (-1.28 – -1.16)
	2	-0.54 (-0.92 – -0.16)	-0.44 (-0.82 – -0.07)

Pneumonia	1	0.69 (0.65 – 0.74)	0.62 (0.58 – 0.65)
	2	0.01 (-0.21 – 0.23)	0.20 (0.00 – 0.40)
Chronic respiratory disease	1	-2.67 (-2.78 – -2.56)	-2.78 (-2.89 – -2.67)
	2	-0.39 (-0.47 – -0.31)	-0.38 (-0.46 – -0.29)
Other respiratory diseases	1	-0.01 (-0.05 – 0.03)	-0.23 (-0.27 – -0.19)
	2	-0.21 (-0.33 – -0.09)	-0.26 (-0.36 – -0.16)
Parkinson's disease	1	-3.30 (-3.44 – -3.16)	-3.34 (-3.50 – -3.18)
	2	-1.64 (-1.73 – -1.54)	-1.97 (-2.08 – -1.86)
Urinary tract infection	1	0.61 (0.51 – 0.70)	0.79 (0.71 – 0.87)
	2	0.23 (0.07 – 0.40)	0.29 (0.18 – 0.40)
Injuries	1	-1.59 (-1.67 – -1.51)	-1.77 (-1.84 – -1.07)
	2	-0.84 (-0.93 – -0.75)	-0.83 (-0.90 – -0.77)
Ill-defined	1	0.42 (0.37 – 0.47)	0.54 (0.51 – 0.58)
	2	0.00 (-0.09 – 0.10)	0.07 (0.00 – 0.14)
**Age**			
50-69		Ref.	Ref.
70-74		0.25 (0.14 – 0.36)	0.12 (-0.01 – 0.24)
75-79		0.28 (0.18 – 0.37)	0.16 (0.05 – 0.27)
80-84		0.28 (0.19 – 0.37)	0.18 (0.07 – 0.28)
85-89		0.31 (0.21 – 0.40)	0.13 (0.02 – 0.23)
90-94		0.30 (0.21 – 0.40)	0.16 (0.06 – 0.27)
95+		0.29 (0.18 – 0.40)	0.22 (0.11 – 0.32)
**Year**			
2006		Ref.	Ref.
2007		0.19 (0.10 – 0.28)	0.12 (0.05 – 0.19)

2008		0.29 (0.21 – 0.38)	0.22 (0.15 – 0.29)
2009		0.25 (0.17 – 0.34)	0.22 (0.15 – 0.28)
2010		0.29 (0.21 – 0.38)	0.30 (0.24 – 0.37)
2011		0.34 (0.26 – 0.43)	0.33 (0.26 – 0.39)
2012		0.39 (0.31 – 0.48)	0.37 (0.30 – 0.43)
2013		0.53 (0.45 – 0.62)	0.49 (0.42 – 0.55)
2014		0.59 (0.51 – 0.67)	0.50 (0.44 – 0.56)
2015		0.56 (0.48 – 0.64)	0.48 (0.41 – 0.54)
2016		0.62 (0.54 – 0.71)	0.55 (0.48 – 0.61)
Constant		0.19 (0.08 – 0.30)	0.60 (0.49 – 0.71)
No.		86,835	142,281

Ref., reference category.

Web Table 5. Regression Results of Dementia UCOD, United States

Variable	Part	Coefficient (95% Confidence Interval)	
		Male	Female
Cause (for each cause, Ref. is "not reported on MCCD")			
IHD	1	-2.72 (-2.73 – -2.70)	-2.94 (-2.95 – -2.93)
	2	-0.22 (-0.24 – -0.20)	-0.28 (-0.29 – -0.27)
Stroke	1	-1.91 (-1.93 – -1.89)	-2.24 (-2.25 – -2.23)
	2	-0.64 (-0.66 – -0.62)	-0.63 (-0.65 – -0.62)
Other heart disease	1	-0.89 (-0.90 – -0.88)	-1.01 (-1.02 – -1.00)
	2	-0.55 (-0.57 – -0.54)	-0.62 (-0.63 – -0.61)
Hypertensive heart disease	1	-1.11 (-1.13 – -1.09)	-1.27 (-1.28 – -1.26)
	2	-0.15 (-0.17 – -0.14)	-0.17 (-0.18 – -0.16)
Other CVD	1	-1.38 (-1.40 – -1.35)	-1.54 (-1.56 – -1.52)
	2	-0.27 (-0.29 – -0.24)	-0.25 (-0.27 – -0.23)
Diabetes	1	-0.92 (-0.95 – -0.89)	-0.94 (-0.96 – -0.92)
	2	-0.24 (-0.25 – -0.22)	-0.25 (-0.26 – -0.23)
Chronic kidney disease	1	-1.18 (-1.21 – -1.15)	-1.18 (-1.21 – -1.16)
	2	-0.34 (-0.36 – -0.31)	-0.31 (-0.33 – -0.29)
Other kidney disease	1	-0.72 (-0.75 – -0.70)	-0.74 (-0.76 – -0.72)
	2	-0.47 (-0.50 – -0.44)	-0.50 (-0.53 – -0.48)
All cancers	1	-3.60 (-3.63 – -3.57)	-3.99 (-4.02 – -3.96)
	2	-0.53 (-0.56 – -0.51)	-0.53 (-0.56 – -0.51)
Sepsis	1	-1.01 (-1.03 – -0.99)	-1.20 (-1.21 – -1.18)
	2	-0.60 (-0.66 – -0.53)	-0.66 (-0.72 – -0.61)

Pneumonia	1	0.43 (0.41 – 0.44)	0.38 (0.37 – 0.39)
	2	-0.20 (-0.25 – -0.16)	-0.26 (-0.3 – -0.23)
Chronic respiratory disease	1	-2.77 (-2.80 – -2.74)	-2.90 (-2.92 – -2.88)
	2	-0.58 (-0.60 – -0.56)	-0.55 (-0.57 – -0.53)
Other respiratory diseases	1	-0.34 (-0.35 – -0.32)	-0.44 (-0.45 – -0.43)
	2	-0.36 (-0.39 – -0.32)	-0.43 (-0.46 – -0.40)
Parkinson’s disease	1	-3.98 (-4.01 – -3.94)	-4.01 (-4.04 – -3.97)
	2	-1.89 (-1.92 – -1.86)	-1.97 (-2.00 – -1.95)
Urinary tract infection	1	0.41 (0.37 – 0.44)	0.62 (0.60 – 0.64)
	2	0.09 (0.05 – 0.14)	0.08 (0.05 – 0.11)
Injuries	1	-1.60 (-1.62 – -1.57)	-1.9 (-1.92 – -1.88)
	2	-1.52 (-1.55 – -1.50)	-1.56 (-1.58 – -1.54)
Ill-defined	1	0.58 (0.56 – 0.59)	0.63 (0.62 – 0.64)
	2	-0.03 (-0.05 – -0.01)	0.01 (-0.01 – 0.02)
**Age**			
50-69		Ref.	Ref.
70-74		0.35 (0.32 – 0.37)	0.32 (0.29 – 0.35)
75-79		0.42 (0.39 – 0.44)	0.38 (0.35 – 0.41)
80-84		0.44 (0.42 – 0.46)	0.40 (0.37 – 0.42)
85-89		0.43 (0.41 – 0.46)	0.40 (0.38 – 0.42)
90-94		0.46 (0.44 – 0.48)	0.42 (0.40 – 0.44)
95+		0.45 (0.42 – 0.48)	0.45 (0.42 – 0.47)
**Year**			
2006		Ref.	Ref.
2007		-0.04 (-0.07 – -0.02)	-0.04 (-0.06 – -0.02)

2008		0.19 (0.16 – 0.21)	0.23 (0.21 – 0.25)
2009		0.21 (0.19 – 0.24)	0.22 (0.20 – 0.24)
2010		0.24 (0.22 – 0.26)	0.27 (0.25 – 0.29)
2011		0.31 (0.28 – 0.33)	0.33 (0.31 – 0.35)
2012		0.33 (0.30 – 0.35)	0.36 (0.34 – 0.38)
2013		0.33 (0.31 – 0.35)	0.39 (0.37 – 0.40)
2014		0.36 (0.33 – 0.38)	0.41 (0.40 – 0.43)
2015		0.35 (0.33 – 0.37)	0.40 (0.38 – 0.42)
2016		0.37 (0.34 – 0.39)	0.43 (0.42 – 0.45)
2017		0.38 (0.36 – 0.40)	0.47 (0.45 – 0.48)
Constant		0.97 (0.94 – 1.00)	1.16 (1.14 – 1.19)
No.		1,141,591	2,096,565

Ref., reference category.

Web Figure 1. International Form of Medical Certificate of Cause of Death (Frame A)

Frame A: Medical Data: Part 1 and 2

		Cause of death	Time interval from onset to death
1 Report disease or condition directly leading to death on line a<br><br>Report chain of events in due to order (if applicable)<br><br>State the underlying cause on the lowest used line	a		
	b	Due to:	
	c	Due to:	
	d	Due to:	
2 Other significant conditions contributing to death (time intervals can be included in brackets after the condition)			

Source: World Health Organization. *International Statistical Classification of Diseases and Related Health Problems, 10th Revision (ICD-10).* (2016 version). Geneva: World Health Organization; 2016.

Web Figure 2. Dementia reported in Part 1 and cardiovascular diseases reported in Part 1 or Part 2 (% of dementia MCOD), by sex, 50+ years, Australia 2006-16 and US 2006-17

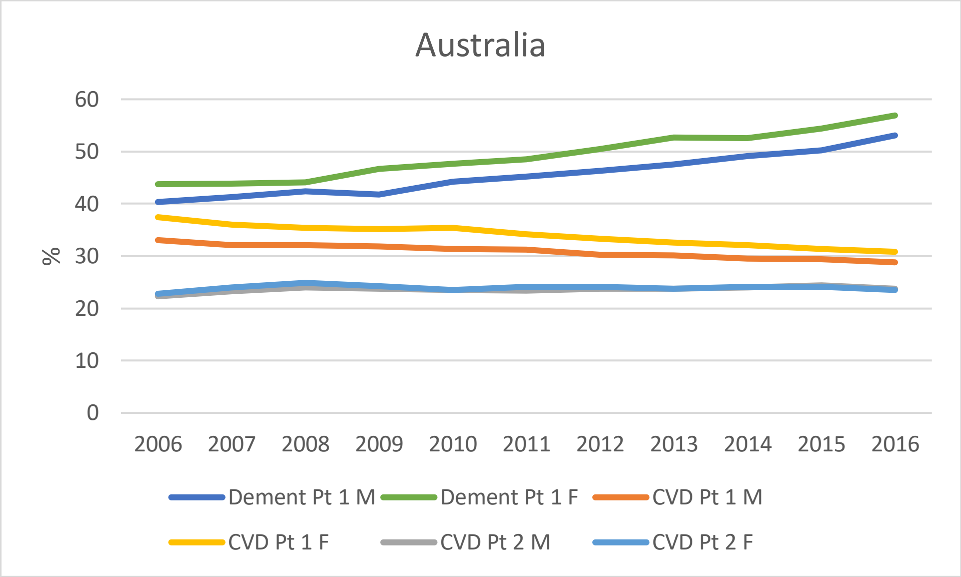

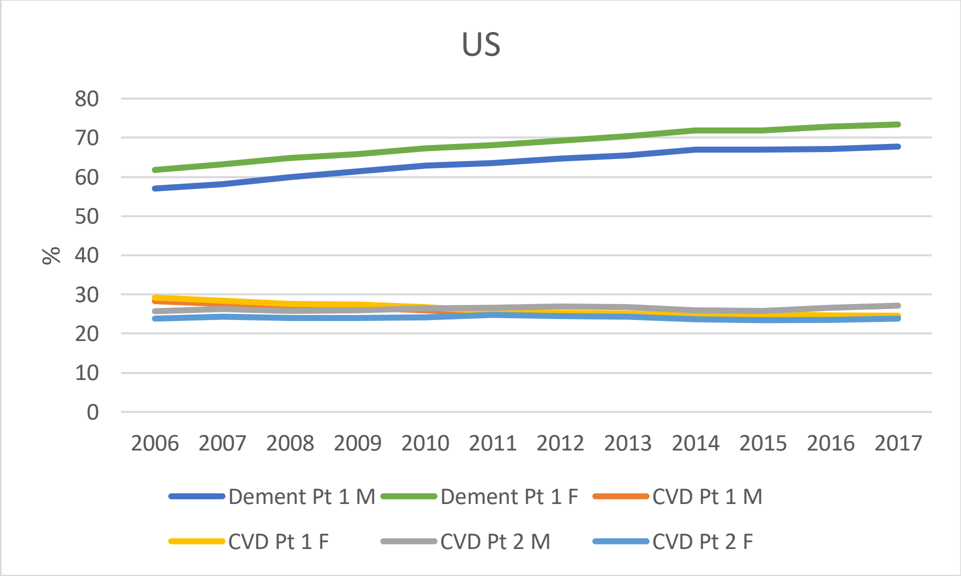

CVD, cardiovascular diseases; Pt, Part.
